# Latent profiles of problematic internet use and their six-month subsequent psychopathology outcomes

**DOI:** 10.1016/j.abrep.2025.100607

**Published:** 2025-04-13

**Authors:** Yi Wang, Brian J. Hall, Yuran Chen, Chun Chen

**Affiliations:** a School of Humanities and Social Science, The Chinese University of Hong Kong, Shenzhen, Guangdong, China; b Center for Global Health Equity, New York University, Shanghai, Shanghai, China

**Keywords:** Problematic internet use, Latent profile analysis, Anxiety, Depression, Stress, Longitudinal study, Rural adolescents

## Abstract

**Objective:**

Problematic Internet Use (PIU) has many adverse effects on youth mental health, including heightened risks of depression and anxiety. However, few studies have systematically investigated the internal heterogeneity of PIU symptoms among rural Chinese adolescents. Data was collected from 5,271 rural Chinese adolescents from two secondary schools in Guizhou and Sichuan at two waves. This study aimed to identify PIU profiles at T1 and examine their relationships with subsequent anxiety, depression, and stress after six months at T2.

**Methods:**

A Latent Profile Analysis (LPA) was conducted to first identify PIU symptom profiles. Then, a “three-step” logistic regression mixed model was conducted to explore the association between PIU patterns and demographic correlates. Anxiety, depression, and stress symptoms collected at the second wave were compared across PIU profiles by using a Bolck-Croon-Hagenaars (BCH) approach.

**Results:**

The study found that (1) The patterns of PIU among rural adolescents could be divided into four subgroups: low PIU group (57.18%), medium PIU group (15.65%), high PIU group (9.01%), and self-blame group (18.16%), which is a uniquely identified group. (2) Being female, an ethnic minority, living off-campus, having left-behind experiences, and having fewer siblings were risk factors for high PIU group membership. (3) The order of severity for anxiety, depression, and stress was as follows: high PIU, medium PIU, self-blame, and low PIU groups. (4) The self-blame group had relatively lower anxiety, depression, and stress scores than the medium PIU group, despite the fact that the self-blame group had higher PIU scores than the medium PIU group, which further strengthens the importance of using a person-centered approach.

**Conclusions:**

Addressing the profiles of PIU is vital for rural Chinese adolescent mental health, necessitating tailored interventions.

## Highlights

• PIU profiles included low PIU, medium PIU, self-blame, and high PIU.
• Female, ethnic minority were risk factors.
• Being off-campus, left-behind status, and fewer siblings were risk factors.
• Psychopathology level ranked from high PIU, medium PIU, self-blame, to low PIU.

## 1 Introduction

The prevalence of problematic internet use (PIU) in China is reported to be notably higher compared to other countries in the world (Guo et al., 2021), with rates reaching up to 23 % (Sun et al., 2020). PIU refers to “excessive or poorly controlled preoccupations, urges, or behaviors regarding the Internet that lead to impairment and distress, which interfere negatively interferences with personal, social, occupational, and/or educational aspects of life” (Baloğlu et al., 2020, Weinstein and Lejoyeux, 2010). PIU is manifested by obsession, neglect, and control disorder (Demetrovics et al., 2008). With the significant disparity in socioeconomic status and mental health resources in rural China (Tang et al., 2018), adolescents in such areas have more likelihood to experience PIU and mental health challenges (Li et al., 2014, Zhu et al., 2019), but most PIU research was conducted among the urban student population. Moreover, previous studies on PIU tended to assume PIU symptoms as homogenous among different individuals, primarily using cross-sectional data in a variable-centered approach. However, these approaches failed to adequately consider individual differences in PIU symptom manifestation and lacked a nuanced investigation of the internal heterogeneity of PIU symptoms within individuals. In light of these limitations, the present study aimed to provide a person-centered examination of PIU among adolescents in rural China through latent profile analysis (LPA) and evaluate the antecedent risk factors and the severity of mental health symptoms on specific profiles.

### 1.1 Understanding PIU from a person-centered approach

With numerous research investigating the characteristics of PIU from a holistic perspective through a variable-centered approach, the individual differences across PIU symptoms are overlooked. These differences are essential for both developing targeted clinical intervention and understanding the intrinsic traits of PIU, particularly in complex rural environments.

Person-centered approach attempts to identify heterogeneous subtypes of individuals through conducting LPA (Morin et al., 2011, Nylund et al., 2007), which concentrates on how individuals differ based on the indicator variable rather than homogenizing the variable across participants (Abar, 2012) and measure the latent level for the probabilities of a person’s membership in various latent profiles (DiStefano and Kamphaus, 2006, Hill et al., 2006). This method captures interindividual variability in intraindividual changes in developmental processes by allowing outcomes to differ between participant subpopulations that are not observed, without presuming population homogeneity (Morin & Litalien, 2019).

Some existing studies have used person-centered approaches to investigate PIU (e.g., Lee et al., 2018). However, few research utilized LPA to explore various symptom patterns of PIU and its influencing factors among Chinese rural adolescents. The present study sought to provide a more nuanced investigation of the manifestation of PIU symptoms and to identify its demographic risk factors in a large group of rural Chinese adolescents.

### 1.2 Demographic factors associated with PIU

Bronfenbrenner’s ecological systems theory suggests that adolescent development is seen as a complex system of relationships including a variety of environmental factors, ranging from the immediate home and school environment to broader cultural values (Bronfenbrenner, 1995, Bronfenbrenner, 1999, Bronfenbrenner and Morris, 1998). However, few studies have ever identified risk and resilience factors of PIU from a comprehensive range of social-ecological demographic variables. This study examined the following demographic variables: age, gender, ethnicity, living-on-campus status, sibling numbers, socioeconomic status (SES), and left-behind status. By identifying how different demographic variables influenced PIU profiles, researchers and practitioners can better tailor PIU prevention and intervention strategies, addressing demographic risk and protective factors unique to the rural cultural settings.

As a key individual characteristic, sex, affected by specific biological factors and social norms (Fattore et al., 2014), plays a crucial role in PIU development. For example, a representative study in Chinese elementary and middle schools found a higher prevalence rate among males (Li et al., 2014). Age, an important indicator of individual development, is also recognized as an important factor influencing Chinese adolescents’ PIU (Zhang et al., 2022).

The microsystem, the environment that individuals directly interact with in their daily lives (e.g., family and school), presents the most direct impact on individuals (Bronfenbrenner & Morris, 1998). A significant microsystem predictor of a child’s psychosocial development is socioeconomic status (SES) (Cabrera et al., 2017). Family structure, particularly the number of siblings, is another crucial microsystem factor that may influence adolescent development and PIU. Research has yielded mixed findings regarding the impact of sibling status on Internet addiction. Yu et al. (2017) found that children with siblings were more likely to become addicted to the Internet. This might be explained by resource dilution theory (Blake, 1981), which suggests that parents’ resources (time, energy, and financial resources) need to be distributed among children, potentially affecting parental supervision of Internet use. However, the relationship between sibling status and adolescent development is complex, particularly in the Chinese context following the One-child policy. While some researchers have found that only children tend to develop certain negative characteristics such as loneliness and narcissism (Cameron et al., 2013). In rural China, individuals with low SES tend to migrate to urban areas in search of better opportunities. According to data from the National Bureau of Statistics of China (2024), the number of migrant workers from rural areas has reached 176.58 million, reflecting a 2.7 % increase. As a result, the phenomenon of left-behind children has emerged. Left behind students are defined as children from rural backgrounds who were raised by grandparents or other family members while their parents moved to an urban location for at least six months (Cheng & Sun, 2015). These left-behind experiences were associated with higher PIU prevalence (Ren et al., 2017), potentially due to reduced parental supervision and emotional support. Along with this situation, another important factor to consider is the policy of boarding schools in rural China, which brings both positive and negative effects on adolescent growth. Living on campus could benefit students through preventing the development of some serious problems, enhancing their competencies such as socializing and independence (Wang et al. 2017), and improving their academic performance and well-being (Liu & Villa, 2020). However, Chinese rural boarding schools frequently fall short of meeting students’ requirements for health, nutrition, and emotional support (Luo et al., 2009). Such a lack of resources in rural schools (e.g., professional mental health staff) might result in more negative outcomes, such as higher depressive symptoms (Zhu et al., 2019), lower self-esteem (Hou et al., 2015) and higher likelihood of bullying (Zhang, 2020).

Building upon the microsystem, the interactions and relationships between different microsystems within the mesosystem can further influence an individual’s growth and development (Bronfenbrenner & Morris, 1998). Long-term labor migration and policies related to boarding schools may reduce family-school interaction (Lu, 2020), yet they also represent distinctive features of adolescent development in rural areas. Therefore, whether students live on campus is also included as a variable. Additionally, the macrosystem consists of political, social, and cultural factors, which shape the environment in which children develop (Bronfenbrenner, 1995, Bronfenbrenner, 1999, Bronfenbrenner and Morris, 1998). China is made up of 56 ethnic groups, including the Han ethnic group as the majority ethnic group comprising 91.11 % (National Bureau of Statistics, 2021) of the Chinese population. Since Chinese ethnic groups are divided by social history, economic life, language, and religion (Maurer-Fazio & Hasmath, 2015), there might be cultural differences among these groups, which could be a significant indicator for PIU. However, most previous PIU studies in China focused on students from the Han ethnic group, while existing yet limited research has found that minority ethnic status was a risk factor for PIU and found the prevalence rate of PIU in students from minority groups was much higher (Lu et al., 2018).

### 1.3 Association between PIU and psychopathology

Adolescents in rural China are particularly vulnerable to mental health challenges, with approximately 20 % at risk for depression and 68 % at risk for various forms of anxiety (Jiang et al., 2022). The pressure from China’s education system, particularly the competitive high school and college entrance exams, intensifies mental health issues, especially in rural areas where resources are limited (Chen, 2022, Chen and Hesketh, 2021). Moreover, rural-to-urban migration has led to many children being raised by grandparents. While the migration income can boost the family’s SES and access to resources, the lack of parental presence can harm the emotional well-being of these left-behind children (e.g., Chen & Zhou, 2021). The interplay of academic stress, limited support, and disrupted family structures presents unique mental health challenges for rural adolescents (Jiang et al., 2022).

Existing studies have investigated the association between PIU and psychopathology including depression and anxiety symptoms, suicidal ideation and problematic behaviors among adolescents (e.g., Huang et al., 2021, Peng et al., 2021, Shang et al., 2024, Zhu et al., 2023), including those in rural China (e.g., Ma & Sheng, 2023). However, inconsistent findings were revealed, particularly on the direction from PIU to psychopathology. For example, Geng and colleagues (2021) found that adolescents with PIU were more likely to develop depression. However, another study also conducted in China found that PIU had no impact on depression, while depression increased the risk of PIU (Zhou et al., 2021). Furthermore, an LPA study also indicated that compared to adolescents in the normal-use category, adolescents in the high PIU and excessive online gaming categories typically had greater depression symptoms and problematic behaviors (Sun et al., 2022). Meanwhile, it should also be acknowledged that PIU might also emerge as a consequence of pre-existing mental health conditions. Existing research has identified potential impacts of psychopathology on PIU, including depression (Ye et al., 2023, Zhou et al., 2021), anxiety (Bernardi & Pallanti, 2009), and impulsivity (Cao et al., 2007). However, findings regarding the impact of PIU on psychopathology remain inconsistent. Moreover, while there have been some studies discussing PIU in rural areas, it remains limited compared to those in urban areas (Pan et al., 2020). Meanwhile, although one study has examined the relationship between PIU and mental health in rural areas through LPA (Sun et al., 2022), it is generally not representative of all rural areas in China due to the difference in developmental level indices and network resource allocation (Li et al., 2014).

### 1.4 The present study

The present study employed a longitudinal design and conducted a person-centered study through LPA to understand PIU patterns among adolescents in rural China. Inspired by Bronfenbrenner’s ecological systems theory, the demographic variables (i.e., age, gender, ethnicity, living-on-campus status, sibling numbers, SES, and left-behind status) were incorporated to investigate the potential antecedent risk factors for PIU. Moreover, this research estimated the subsequent impacts of PIU on experiences with depression, anxiety, and stress. Overall, we aimed to (1) examine PIU latent profiles at the beginning of an academic year in 2022 (T1), (2) the association between demographic variables at T1 and PIU profiles at T1, and (3) the association between PIU profiles at T1 and subsequent psychopathology symptoms in 2023 (T2) among Chinese rural adolescents.

## 2 Materials and methods

### 2.1 Participants and procedure

The present study is part of a larger project focusing on adolescent mental health and resilience in rural China. Data were collected at two-time points (T1: October 2022; T2: April 2023) from two boarding schools located in rural areas of Guizhou and Sichuan Provinces, China. Collaboration with these schools was facilitated through a non-profit organization dedicated to supporting education for students from low-income rural areas. After being informed about the project, both schools voluntarily agreed to participate in the study. Prior to data collection, written informed consent was obtained from both participants and their guardians. In the informed consent form, we stated that all information provided during the study would remain confidential, and any results published or disseminated from the research would only be presented as aggregated statistical data and be fully anonymous. Data was collected during regular school hours, and the survey took around 15  min. Participants were invited to complete a web-based questionnaire via the Credamo platform under the supervision of homeroom teachers and psychology teachers. Given that the study addressed sensitive topics such as depression, local mental health hotlines were provided to the participants and their guardians. This study was reviewed and approved by the Ethics Committee of [blinded for review] (No. EF20220602002).

At T1, a total of 9,097 middle and high school students participated in the study. At T2, upon matching data with T1, 5,271 students (M = 14.82, SD = 14.038, 56 % females) remained, yielding a retention rate of 57.94 %. 3,498 (66.4 %) students were from ethnic minorities, and the majority of the sample (94.5 %, n = 4,981) lived on campus. There were 1,628 (31 %) participants who reported having at least one parent as a migrant worker (left-behind status). The mean of family socioeconomic status (SES) was 2.59 (SD = 1.639), indicating a relatively low level of status. More details are presented in Table 6.

### 2.2 Measures

#### 2.2.1 Problematic internet use questionnaire short form (PIUQ-SF)

We measured PIUQ at T1. The PIUQ has proven a valid and reliable instrument for measuring PIU symptoms (Laconi et al., 2019). A short version with nine items on three subscales, including obsession (e.g., “How often do you feel tense, irritated, or stressed if you cannot use the internet for as long as you want to?”), neglect (e.g., “How often do you neglect household chores to spend more time online?”), and control disorder (e.g., “How often do you try to decrease the amount of time you spend online but fail?”), was developed by Demetrovics et al. (2008) as a second-order three-factor model structure with three items within each factor. PIUQ-SF uses a 5-point Likert scale (range from “never” to “always/almost always”) to estimate the frequency of PIU symptoms. The Chinese version of the PIUQ-SF was translated and validated by Koronczai et al. (2017). The Cronbach’s alpha of the measure in the present sample is 0.87 at T1.

#### 2.2.2 The depression anxiety stress scale 21 (DASS-21)

We measured DASS-21 at T1 and T2. The 21-item Depression Anxiety Stress Scale (DASS; Lovibond & Lovibond, 1995) version is a self-report measure of differentiation between depression (e.g., “I felt that life was meaningless”), anxiety (e.g., “I felt scared without any good reason”), and stress-related mood disorders (e.g., “I found it difficult to relax”). The DASS-21 is a 4-point Likert scale from “does not apply to me at all” to “completely applies to me”. The validity and reliability of the Chinese version of DASS-21 have been demonstrated in Chinese adolescents (Gong et al., 2010). Cronbach’s alpha was 0.90 for depression, 0.87 for anxiety, and 0.87 for stress at T2.

#### 2.2.3 Demographic variables

Participants were asked to answer demographic questions including age, gender, ethnicity, residency status, sibling numbers, SES, and left-behind status. Ethnicity was collected because the two counties are multi-ethnic areas. The residence status is to collect whether they live on campus or at home. The Family Affluence Scale (FAS; Hobza et al., 2017) questionnaire was administered to measure participants’ SES. In the present sample, the Cronbach’s alpha for FAS was 0.73. The psychometric properties have been empirically validated among Chinese adolescents in previous study (Liu et al., 2012). Left-behind status was determined by asking if their father or mother had migrated to another city for work in the previous year and had not returned home for more than six months. Responses were coded as 0 (neither parent absent), 1 (one parent absent), or 2 (both parents absent), allowing us to capture different levels of parental absence due to work migration.

### 2.3 Statistical analyses

To determine the pattern of PIU among participants, we conducted an LPA three-step approach using Mplus 8 (Muthén and Muthén, 2017, Nylund-Gibson et al., 2019) to investigate heterogeneity and identify the most mutually exclusive classes in PIU and risks of demographic variables and distal outcomes.

First, prior to conducting LPA on the nine PIUQ-SF items at T1 to determine the optimal number of profiles, we examined the normality of the distributions. LPA assumes population heterogeneity (Spurk et al., 2020). The distributions of the PIUQ-SF items were examined, with skewness values ranging from 1.536 to 1.995 and kurtosis values from 2.420 to 3.935. These moderate deviations from normality are typical for Likert-scale data. LPA is robust to moderate skewness and kurtosis, especially with large sample sizes. Therefore, the original data were used without transformation, ensuring interpretability. Second, we conducted LPA. Models with one to five classes were estimated and compared using multiple fit indices: Akaike Information Criterion (AIC), Bayesian Information Criterion (BIC), sample size adjusted BIC (saBIC), and Lo-Mendell-Rubin likelihood ratio test (LRT). The Lo-Mendell-Rubin likelihood ratio test (LRT) examines whether a k-class model fits significantly better than a (k-1)-class model, with a significant p-value (<.05) indicating the k-class model is preferred. The Bayes Factor (BF) represents the odds ratio of adjacent models, with values below 1 supporting the more complex model. Model selection was based on: (a) statistical fit indices, (b) entropy values (criterion > 0.80), (c) average latent class probabilities (criterion > 0.70), (d) profile size (ensuring no class contained less than 5 % of the sample).

Second, after identifying the best model, we examined how demographic variables (gender, age, ethnicity, residence status, SES, left-behind status, and number of siblings) influence profile membership by using multivariable logistic regression. Prior to regression analysis, multicollinearity was assessed through Variance Inflation Factors (VIF), with all values below 2 indicating no serious multicollinearity concerns (Table 4).

Third, we examined how these profiles were associated with subsequent psychopathology (anxiety, depression, and stress at T2) using the Bolck-Croon-Hagenaars (BCH) approach, which maintains the original latent profiles while accounting for classification uncertainty (Vermunt, 2010). The BCH method uses weighted multiple group analysis when testing profile differences in distal outcomes. Effect sizes for mean differences were calculated using Cohen’s d, with values of 0.2, 0.5, and 0.8 representing small, medium, and large effects respectively. Attrition analyses compared participants completing only T1 PIU measures to those completing both T1 and T2 PIU. Significant differences were observed between the two groups (t (9071) = -7.128, *p* < 0.001). However, the Little’s MCAR test confirmed that the missing data were missing completely at random (χ2 (1) = 1.276e-31, p = 1.000), indicating minimal attrition bias. Descriptive statistics are in Table 1. The data and analysis code that support the findings of the study are available on request from the corresponding author.

**Table 1:** Correlation Coefficients among variables.

	Mean/Proportion	SD	1	2	3	4	5	6	7	8	9	10	11
Gender	0.56	0.496	1										
Age	14.82	14.038	−0.016	1									
Ethnicity	0.75	0.602	−0.025	0.020	1								
Living on-campus or not	0.95	0.227	0.061**	0.002	0.027	1							
Sibling numbers	1.59	0.890	0.088**	0.004	0.312**	0.064**	1						
SES	2.59	1.639	0.027*	−0.011	−0.267**	−0.064**	−0.286**	1					
Left behind	0.46	0.746	0.000	0.027	0.308**	0.045**	0.048**	−0.203**	1				
Anxiety	9.33	10.023	0.046**	0.016	0.176**	−0.006	−0.085**	−0.031*	0.213**	1			
Depression	9.17	10.111	0.031*	0.010	0.157**	−0.008	−0.077**	−0.050**	0.177**	0.893**	1		
Stress	10.68	10.284	0.072**	0.026	0.200**	−0.005	−0.083**	−0.046**	0.232**	0.909**	0.888**	1	
PIU	1.86	0.73	0.070**	0.015	0.029*	−0.035*	−0.074**	−0.008	0.114**	0.322**	0.327**	0.347**	1

*p < 0.05, ** *p* < 0.01.

## 3 Results

### 3.1 Descriptive statistics

The descriptive data and correlation analysis on PIU at T1 and psychopathology (i.e., depression, anxiety, and stress) at T2 were presented in Table 1. Results showed that PIU at T1 had significant positive correlations with anxiety (*r* = 0.322, *p* < 0.01), depression (*r* = 0.327, *p* < 0.01), and stress (*r* = 0.347, *p* < 0.01) at T2, suggesting moderate associations between PIU and psychological distress. The moderate strength of these correlations is particularly meaningful in the rural context, where mental health resources are limited, and internet use may serve as both a coping mechanism and a potential risk factor. Additionally, the significant correlations between left-behind status at T1 and psychological outcomes at T2 (ranging from *r* = 0.177 to 0.232, *p* < 0.01) highlight the compound challenges faced by rural adolescents, where parental absence may exacerbate both PIU and mental health issues.

### 3.2 Latent profile analysis of PIU

Considering the optimal fit statistics, parsimony, category significance, the minimum number of participants in the smallest class, and model comparison, the four-class model was selected based on a total of 9 items measured at T1. as shown in Table 2 and Fig. 2. The four-class model showed significant improvement over the three-class model (LRTS = 2148.04, *p* < 0.001), while the five-class model, although showing better fit (LRTS = 9060.28, *p* < 0.001), produced an impractically small class (3.01 % of the sample). This four-class model showed good classification quality (entropy = 0.895) and high average posterior probabilities (ranging from 0.886 to 0.969). Each class contained more than 5 % of the sample, with the smallest class comprising 9.01 % of participants.

**Table 2:** Fit indices of LCA Class Models.

**Model (K-Class)**	**LL**	**npar**	**AIC**	**CAIC**	**BIC**	**saBIC**	**AWE**	**LRTS**	**BF (K, K + 1)**	**Entropy**	**Number of minimal class/percentages**
1-class	−67827.111	18	135690.22	135826.48	135808.48	135751.28	136016.74	–	0.000	−	−
2-class	−60453.979	28	120963.96	121175.92	121147.92	121058.94	121471.88	14746.26	0.000	0.927	1208/22.91 %
3-class	−58056.156	38	116188.31	116475.97	116437.97	116317.22	116877.63	4795.65	0.000	0.878	580/11 %
4-class	−56982.137	48	114060.27	114423.63	114375.63	114223.10	114930.99	2148.04	0.000	0.895	475/9.01 %
5-class	−52451.997	58	105019.99	105459.05	105401.05	105216.75	106072.11	9060.28	0.000	0.953	159/3.01 %

**Table 3:** Coefficients and odds ratio for the four-class model with demographic variables.

**Variables**		**Gender (reference group = male)**	**Age**	**Ethnicity**	**Living at school or not (reference group = not)**	**SES**	**Left-Behind Status**	**Sibling Numbers**

C1vs C4	B	−0.186	−0.004	−0.213*	0.435*	0.041	−0.327**	0.301**
	SE	0.102	0.017	0.091	0.206	0.032	0.069	0.06
	Odds Ratio	0.83*	0.996	0.809**	1.545	1.042	0.721**	1.351**
C2 vs C4	B	−0.008	0	−0.191	0.068	0.048	0.078	0.163*
	SE	0.119	0	0.104	0.236	0.038	0.077	0.069
	Odds Ratio	0.992	1	0.826*	1.07	1.049	1.081	1.176*
C3 vs C4	B	0.178	0	−0.075	0.014	0.015	−0.044	0.176**
	SE	0.117	0	0.103	0.23	0.036	0.076	0.067
	Odds Ratio	1.195	1	0.928	1.015	1.015	0.957	1.193*
C2 vs C1	B	0.178*	0.003	0.022	−0.367*	0.007	0.404**	−0.139**
	SE	0.081	0.017	0.075	0.17	0.027	0.055	0.049
	Odds Ratio	1.195*	1.003	1.022	0.693**	1.007	1.499**	0.871**
C3 vs C1	B	0.364**	0.004	0.138	−0.421**	−0.026	0.283**	−0.125**
	SE	0.078	0.017	0.073	0.162	0.025	0.054	0.046
	Odds Ratio	1.44**	1.004	1.147	0.657**	0.974	1.327**	0.883**
C3 vs C2	B	0.186	0.001	0.116	−0.053	−0.033	−0.122	0.014
	SE	0.099	0	0.089	0.197	0.032	0.064	0.059
	Odds Ratio	1.205	1.001	1.123	0.948	0.968	0.886*	1.014

**p < 0.01, *p < 0.05; C1 = Low PIU, C2 = Medium PIU, C3=Self-Blame, C4=High PIU.

**Table 4:** VIF and 95% confidence intervals.

Variables		Gender (reference group = male)	Age	Ethnicity	Living at school or not (reference group = not)	SES	Left-Behind Status	Sibling Numbers
VIF		1.08	1.12	1.31	1.06	1.18	1.22	1.24
C1vs C4	CI	0[.673, 1.025]	0[.962,1.032]	0[.673, 0.974]	[1.234, 1.951]	0[.981, 1.105]	0[.612, 0.848]	[1.202, 1.518]
C2 vs C4	CI	0[.796, 1.237]	[1.000,1.000]	0[.67, 1.018]	0[.673, 1.352]	0[.972, 1.129]	0[.915, 1.271]	[1.051, 1.316]
C3 vs C4	CI	0[.964, 1.483]	[1.000,1.000]	0[.747, 1.152]	0[.684, 1.506]	0[.942, 1.093]	0[.821, 1.115]	[1.062, 1.341]
C2 vs C1	CI	[1.021, 1.399]	0[.969,1.038]	0[.884, 1.183]	0[.573, 0.832]	0[.954, 1.063]	[1.334, 1.689]	0[.812, 0.933]
C3 vs C1	CI	[1.228, 1.689]	0[.969,1.040]	0[.963, 1.363]	0[.573, 0.761]	0[.926, 1.025]	[1.146, 1.544]	0[.789, 0.978]
C3 vs C2	CI	0[.993, 1.462]	[1.000,1.002]	0[.947, 1.333]	0[.681, 1.318]	0[.907, 1.031]	0[.875, 1.112]	0[.898, 1.146]

**Table 5:** Means of anxiety, depression, and stress in each class.

**PIU Class**	**Anxiety** **(Range 0–42)**	**SE**	**Depression** **(Range 0–42)**	**SE**	**Stress** **(Range 0**–**42)**	**SE**
Class 1: Low PIU	6.778_a_	0.175	6.647_a_	0.178	7.878_a_	0.182
Class 2: Medium PIU	13.201_b_	0.404	12.960_b_	0.406	14.720_b_	0.403
Class 3: Self-Blame	10.377_c_	0.355	9.958_c_	0.357	12.190_c_	0.353
Class 4: High PIU	16.462_d_	0.541	16.789_d_	0.524	18.157_d_	0.522

*Note*. Means that do not share the same subscripts differ at p < 0.001.

**Table 6:** The descriptive statistics.

**Item**	**Category**	**Number**	**Percentage**
Gender	Male	2302	56.33 %
	Female	2969	43.67 %
Ethnicity	Han	1773	33.64 %
	Yi	3035	57.58 %
	Others	463	8.78 %
Living Status	Living at school	4983	94.54 %
	Living at home	288	5.46 %
Left Behind Status	No	3643	69.11 %
	One parent out	816	15.48 %
	Both parents out	812	15.41 %
Sibling Numbers	0	402	7.63 %
	1	2428	46.06 %
	2 and more	2441	46.31 %

**Fig. 1:**
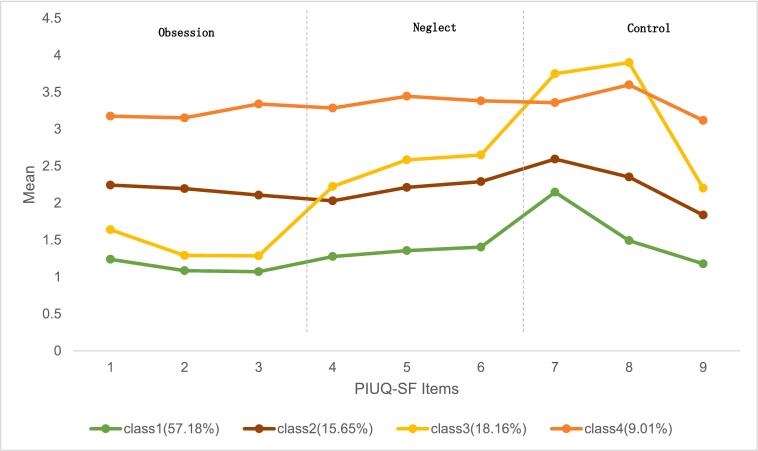
*Four-Class Model. Note*. 1 = feel tense, irritated, or stressed if cannot use the internet. 2 = feel tense, irritated, or stressed if cannot use the internet for days. 3 = feel depressed when not on the internet and feeling stop once back online. 4 = neglect household chores to spend more time online. 5 = spend time online when you’d rather sleep. 6 = people complain about you spending too much time online. 7 = try to decrease the amount of time spent online. 8 = wish to decrease the amount of time spent online but do not succeed. 9 = try to conceal the amount of time online. Class 1 = Low PIU, Class 2 = Medium PIU, Class 3=Self-Blame, Class 4=High PIU.

**Fig. 2:**
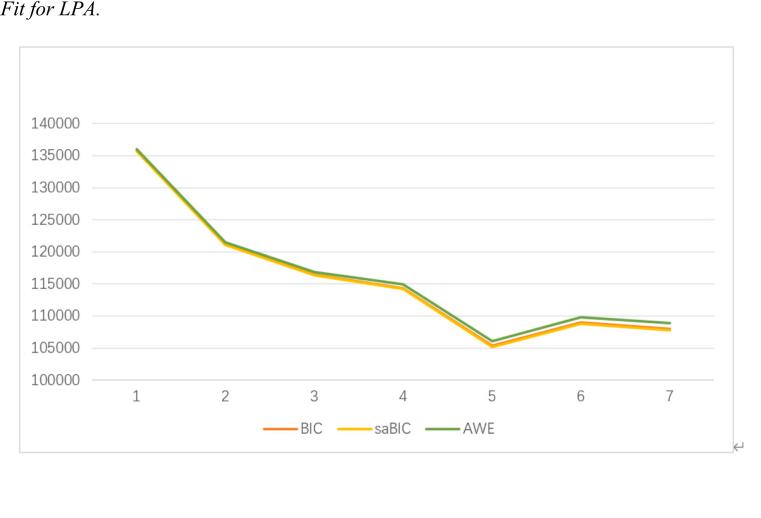
Fit for LPA.

The representation in the four-class model was (1) Class 1: low PIU (PIU mean = 1.36), accounting for 57.18 % of the sample; students in this class scored relatively low on all items; (2) Class 2: medium PIU (PIU mean = 2.21), accounting for 15.65 % of the sample; students in this class scored in the middle of the range on all items; (3) Class 3: self-blame group (PIU mean = 2.39), students who showed lower addiction score (the second lowest item 1,2,3 indicated that they did not feel excessive anxiety, depression, or mood swings when they are unable to access the Internet), medium neglect score (the second highest item 4,5,6), and higher control disorder score (the highest scores on item 7: attempting to reduce internet use, and item 8: wanting to reduce but failing, and the second highest item 9: try to conceal the amount of time online) account for 18.16 % of the total; and (4) Class 4: high PIU group (PIU mean = 3.32), accounting for 9.01 %; students in this class scored relatively high on all items. Students in this class scored high across all items, reflecting severe neglect, obsession, and loss of control. The four-class model is presented in Fig. 1.

Class 3 was named the “self-blame group”, because participants had higher self-blame scores on the dimension of control disorder (the highest item7,8 and the second highest item 9 suggested that they tended to be aware that they spend too much time online and had a strong desire to reduce their use of the Internet). These individuals expressed a belief that they should reduce their online time but consistently felt they were unable to do so, and even though their addiction scores were low. This group exhibited high self-blame and control scores, suggesting a unique cognitive-emotional pattern of attributing internet use problems to personal failings while maintaining strong self-regulation.

### 3.3 Demographic factors associated with PIU latent profiles

The likelihood of being placed in a particular class differed among different demographic variables. According to McFadden's pseudo R-squared (0.091) and adjusted R-squared (0.089), the model explains approximately 8.89 % of the variance in the dependent variable, indicating a relatively modest explanatory power. As shown in Table 3 and Table 4, compared with Class 4, students who were Han ethnicity, living on campus, accompanied by both parents and more siblings, were more likely placed in Class 1 (ethnicity: b = -0.21, *p* < 0.05, OR = 0.81, 95 % CI = [0.673, 0.974]; staying on-campus or not: b = 0.44, *p* < 0.05, OR = 1.55, 95 % CI = [1.234, 1.951]; left-behind: b = -0.33, *p* < 0.01, OR = 0.72, 95 % CI = [0.612, 0.848]; sibling numbers: b = 0.301, *p* < 0.01, OR = 1.35, 95 % CI = [1.202, 1.518]). Being male, living on campus, their parents not leaving them behind, and having more siblings were more likely to be in Class 1 compared with Class 2 and Class 3 (C2 VS C1: sex: b = 0.18, *p* < 0.05, OR = 1.20, 95 % CI = [1.021, 1.399]; staying on-campus or not: b = -0.37, *p* < 0.05, OR = 0.69, 95 % CI = [0.573, 0.832]; left-behind: b = 0.40, *p* < 0.01, OR = 1.50, 95 % CI = [1.334, 1.689]; sibling numbers: b = -0.14, *p* < 0.01, OR = 0.87, 95 % CI = [0.812, 0.933]; C3 VS C1: sex: b = 0.36, *p* < 0.01, OR = 1.44, 95 %=[1.228, 1.689]; staying on-campus or not: b = -0.42, *p* < 0.01, OR = 0.66, 95 % CI = [0.573, 0.761]; left-behind: logit = 0.28, *p* < 0.01, OR = 1.33, 95 % CI = [1.146, 1.544]; sibling numbers: b = -0.13, *p* < 0.01, OR = 0.88, 95 % CI = [0.789, 0.978]).

### 3.4 PIU latent profiles associated with depression, stress, and anxiety

Six months after T1, anxiety, depression, and stress levels were measured at T2 to serve as distal outcomes associated with the PIU latent profiles. Overall, students in Class 4 had the highest values of depression, anxiety, and stress. The mean differences across the four classes were also observed similarly on depression, anxiety, and stress, as shown in Table 5. All four classes showed significant psychopathology differences with each other. Class 4 prevalently had the highest depression, anxiety, and stress scores (depression: *M* = 16.79; anxiety: *M* = 16.46; stress: *M* = 18.16). Class 2 showed the second-highest depression, anxiety, and stress scores (depression: *M* = 12.96; anxiety: *M* = 13.20; stress: *M* = 14.72). Following Class 2, Class 3 showed the third highest depression, anxiety, and stress scores (depression: *M* = 9.96; anxiety: *M* = 10.38; stress: *M* = 12.19). Class 4 showed the lowest depression, anxiety, and stress scores (depression: *M* = 6.65; anxiety: *M* = 6.78; stress: *M* = 7.88).

The group differences are presented below and in Table 5. For depression, the difference between Class 1 and Class 2 is *p* < 0.001, d = -6.31; the difference between Class 1 and Class 4: *p* < 0.001, d = -10.14; the difference between Class 3 and Class 4: *p* < 0.001, d = -6.83; the difference between Class 2 and Class 3: *p* < 0.001, d = 3.00. For anxiety, the difference between Class 1 and Class 2 is *p* < 0.001, d = -6.42; the difference between Class 1 and Class 4: *p* < 0.001, d = -9.68; the difference between Class 3 and Class 4: *p* < 0.001, d = -6.09; the difference between Class 2 and Class 3: *p* < 0.001, d = 2.82. For stress, the difference between Class 1 and Class 2 is *p* < 0.001, d = -6.84; the difference between Class 1 and Class 4: *p* < 0.001, d = -10.28; the difference between Class 3 and Class 4: *p* < 0.001, d = -5.97; the difference between Class 2 and Class 3: *p* < 0.001, d = 2.53.

Cohen’s d confirmed the significance of these differences, with effect sizes ranging from small (e.g., d = -0.245, −0.261, −0.217 for anxiety, depression and stress between Class 3 and Class 2) to moderate (e.g., d = 0.577, 0.657, 0.575 for anxiety, depression and stress between Class 4 and Class 3) and large (e.g., d = -1.007, −1.045, −1.042 for anxiety, depression and stress between Class 1 and Class 4).

## 4 Discussion

This study conducted LPA to investigate the PIU profiles at one time point and examined its relationship with a range of social-ecological demographic variables and psychopathology in rural Chinese adolescents. A four-class PIU pattern was identified into low PIU, medium PIU, self-blame, and high PIU group. The findings also suggested that demographic variables played an essential role in distinguishing different patterns of PIU among rural Chinese adolescents. In addition, different PIU profiles were associated with anxiety, depression, and stress symptoms. The results revealed the heterogeneity of adolescent PIU in a rural Chinese context and identified its risk and protective factors and outcomes.

### 4.1 Descriptive findings

Our analysis revealed moderate associations between T1 Problematic Internet Use (PIU) and T2 psychological distress (depression, anxiety, and stress). We also found significant correlations between T1 left-behind status and T2 psychological outcomes. These findings have important implications for rural Chinese adolescents. First, the moderate correlation between PIU and psychological distress suggests that monitoring internet use patterns could help identify potential mental health issues early, especially in areas lacking professional psychological services. Second, internet use plays a complex dual role in rural settings. While it provides valuable information and social support resources that may not be available locally, excessive use as an emotional escape could prevent adolescents from developing healthier coping strategies. Third, the link between left-behind status and poor psychological outcomes highlights the need for comprehensive interventions. These interventions should address both internet use management and social support needs, particularly for adolescents with migrant parents.

### 4.2 Latent profiles of PIU

The classifications of PIU identified were mostly consistent with previous studies of LPA in rural Chinese adolescent samples by Sun (2022), where they identified normal internet use, low internet addiction, high internet addiction, and overuse of online games. The distinction from previous findings is the identification of the special group, the self-blame group. The unique finding of the self-blame group in our study accounted for almost one in five students. This group exhibited distinctive behavioral and cognitive patterns, such as lower obsession scores but higher self-blame and control disorder scores. This group of students demonstrated high self-expectation. Although the results showed that they wanted to reduce the frequency of their Internet access, their obsession scores were relatively low. These adolescents reported feeling guilty after excessive internet use and expressed a desire to reduce their online time, indicating heightened self-awareness and self-regulation.

The self-blame group’s behavior may also be rooted in the socio-cultural and economic context of rural China. It is possible that even though this group of adolescents wished to manage their online time, in reality, rural adolescents had fewer extracurricular activities compared with urban counterparts (Zhang & Tang, 2017). Consequently, the Internet has become a medium that satisfies their experience and exploration needs and functions as an escape or avoidance mechanism (Lowry et al., 2015). Additionally, previous research has shown that rural adolescents' educational expectations are generally lower (Chang et al., 2016). Lower educational expectations are often accompanied by lower academic self-efficacy (Ansong et al., 2019, Michael, 2019), and this deficient self-view might lead to excessive self-criticism when failing to control their Internet use.

This study validated the profiles of PIU within the rural context. At the same time, our findings innovatively introduced a previously unidentified ‘self-blame’ group, shedding light on a unique psychological profile. This research not only enhanced the understanding of PIU from a person-centered perspective but also provided a crucial foundation for the development of future intervention strategies in rural regions.

### 4.3 Demographic factors associated with PIU latent profiles

Bronfenbrenner’s ecological systems theory (1995, 1999) emphasizes that adolescent development is shaped by multiple environmental systems. As expected in this study, the variables proposed based on the theory played a significant role in predicting different profiles. At the mesosystem level, our findings implied that boarding school environments offered structured routines that limit internet use, while the educational and cultural barriers faced by minority students contributed to exacerbating PIU. Notably, the identification of the self-blame profile highlighted how the interaction between cultural values (macrosystem) and individual characteristics might give rise to unique patterns of internet use, patterns not previously observed in urban or Western samples.

Our results showed that being female, an ethnic minority, living off-campus, having left-behind experiences, and having fewer siblings were risk factors for being in a higher PIU group. Consistent with some studies (e.g., Gansner et al., 2019), males were more likely to be in the low PIU group (versus medium and self-blame group), which means female students were more vulnerable to PIU and more likely to have control disorder problems. This could be explained by the fact that in rural areas, females had less safe space to outspeak their needs. They were more likely to be mistreated because of sex discrimination (Hannum et al., 2009). Therefore, the Internet gives them a place to escape, further leading to PIU and control issues. This is particularly concerning given that female students in rural areas face unique challenges including gender-based educational discrimination and restricted social activities. To address these gender disparities, schools should implement gender-sensitive PIU prevention programs that provide alternative recreational activities specifically targeting female students' interests and needs.

Ethnic minority students were more likely to be classified in the high PIU group, which might be explained by cultural differences. In Han ethnic families, parents tended to be relatively strict with their children as the Preferential Admission Policy gives bonus points in national college entrance exams to students with minority ethnicities (Wang, 2007). Moreover, with the Han culture being localized in different regions in China, it was likely that students from ethnic minority backgrounds would experience struggle in school settings as the main teaching language is Mandarin (Yang et al., 2015), further resulting in their seeking online spaces to avoid these frustrations (Li et al., 2015).

Students who lived on campus were more represented in the Low PIU group than in the other three groups. This suggested that living on campus could effectively reduce PIU. In rural China, schools had a strict residential system with precise light-out times and internet and cellphone control. Hence, the strict on-campus schedule and cell phone ban policy prevented many students from obtaining higher PIU scores, suggesting that restrictions on Internet access time may effectively reduce PIU among rural adolescents.

Moreover, parental presence was a protective factor. Compared with adolescents who had one or both parents left behind, children whose parents did not migrate were more likely to be classified in the low PIU group. Being at home, parents would be able to better manage their children's Internet problems. Having more emotional ties would also reduce the dependence on the Internet. The presented study also showed that the greater number of siblings was a protective factor against PIU. This was because more siblings could make up for the lack of parental companionship. Siblings in the same generation could confide in and help each other to effectively reduce PIU.

### 4.4 PIU latent profiles associated with psychopathology

This study revealed that Chinese rural adolescents with distinct profiles of PIU exhibited significantly varied levels of anxiety, depression and stress symptoms. Participants in the self-blame group, high PIU group, and medium PIU group displayed more severe anxiety, depression, and stress symptoms than those with low PIU. In particular, Class 4 (High PIU) showed significantly higher psychopathology scores compared to Class 1 (Low PIU). This result suggested that high PIU was accompanied by high psychological distress in adolescents from rural areas, consistent with prior research (Amorosi et al., 2012, Dalbudak et al., 2013). The substantial difference may indicate that PIU is not merely a statistical phenomenon but has meaningful psychological consequences for rural adolescents. High levels of PIU may serve as an early warning signal for adolescent psychological distress, and timely identification and intervention of excessive internet use behaviors may help prevent the development of psychological distress. Meanwhile, high levels of PIU may lead to increased anxiety, depression and stress, and conversely, anxiety, depression, and stress may drive adolescents to use the internet more as a way to escape reality. This bidirectional relationship may create a vicious cycle. Therefore, when assessing adolescent psychological problems, attention also should be paid to their internet use patterns. Several mechanisms may explain why higher PIU leads to increased psychological distress. First, social withdrawal serves as an important mediating mechanism between PIU and psychological distress, accounting for 9.4 %-29.0 % of their association (Narita et al., 2025). Excessive internet use often diminishes face-to-face communication, interpersonal relationships and social support (Kim, 2017), exacerbating psychological distress such as anxiety, depression, and stress symptoms. This creates a vicious cycle where adolescents increasingly retreat to online spaces to cope with their distress, further exacerbating their anxiety and depression symptoms. Secondly, the family environment plays a crucial role in the development of PIU (Casaló and Escario, 2019). Research has demonstrated that negative family environments characterized by low expressiveness and high conflicts contribute to PIU both directly and indirectly, which also increase adolescents' depression levels (Sela et al. 2020).

Interestingly, this study revealed that participants in the self-blame group exhibited less anxiety, depression, and stress symptoms than those in the medium PIU group, despite having higher PIU scores than the medium PIU groups. The self-blame group had the highest control disorder score and the second high neglect score, and the total PIU mean score was higher than the medium PIU group. This group’s high control scores may act as a psychological buffer, mitigating the emotional toll of PIU. Additionally, the relatively low obsession scores in this group suggest that obsession with the internet, rather than PIU severity alone, plays a critical role in influencing psychological distress. A literature review indicates that similar to how individuals use substances like alcohol, people seek temporary escape from reality through internet addiction as an avoidance strategy (Melodia et al., 2020). This reveals that avoidance strategies are one of the key factors leading to internet obsession. The findings highlight the importance of targeted interventions tailored to the unique needs of each group.

### 4.5 Practical implications

Our findings informed the need to develop ecologically-informed interventions targeting multiple system levels to address PIU among rural Chinese adolescents. First, at the individual level (microsystem), schools should consider integrating PIU as a screening measure within school-wide mental health assessments. Given that PIU is a significant indicator of psychopathology and maladaptive behaviors among students, PIU progress monitoring can provide valuable insights into student mental wellbeing (D'Angelo & Moreno, 2020). Notably, our research findings from the self-blame group (class 3) revealed that the relatively low obsession scores in this group suggest that obsession with the internet, rather than PIU severity alone, plays a critical role in influencing psychological distress, with avoidance strategies being one of the key factors leading to internet obsession (Melodia et al., 2020). Therefore, for students in the high PIU group (Class 4), intensive individual interventions such as cognitive behavioral therapy should focus on enhancing self-control, emotional regulation skills, and improving face-to-face social interactions (Ke & Wong, 2018). Interventions should pay special attention to female students who showed higher vulnerability to PIU. Schools should consider implementing gender-sensitive programs that provide safe spaces for female students to express their needs and develop healthy coping strategies, given that they may face unique challenges in rural settings, including gender-based discrimination (Hannum et al., 2009).

At the microsystem level (family and school), our findings highlight several key intervention targets. For family-based interventions, special attention should be paid to left-behind children, as our results showed they were more likely to develop PIU. Programs should be developed to strengthen guardian-child relationships and enhance communication between migrant parents and their children (Sun et al., 2015). For example, schools could regularly organize “family workshops” and promote the importance of maintaining online communication between migrant parents and their children.

Given our finding that having more siblings served as a protective factor against PIU, we recommend that intervention programs include a peer support component to mitigate PIU among youth (Choo et al., 2020). Schools could establish peer support networks for only children or those separated from siblings to provide similar social benefits.

The boarding school environment (another crucial microsystem) showed protective effects against PIU in our study. Schools should leverage this advantage by implementing structured schedules and supervised internet access policies (Zhao et al., 2017), while providing social support activities such as school group counseling and interest groups.

At the mesosystem level, interventions should address the reduced family-school interaction caused by long-term labor migration and boarding school policies (Zhang & Wu, 2020). Schools could establish regular virtual communication channels between migrant parents and teachers, ensuring continued parental involvement despite physical distance.

At the macrosystem level, our findings about ethnic minority students' higher vulnerability to PIU demonstrate the need for nurturing multicultural environments. Therefore, we should promote multicultural environments in schools to help teachers improve their cultural adaptability and develop cultural sensitivity, thereby enhancing teacher-student relationships and reducing PIU among ethnic minority students (Glock et al., 2019).

## 5 Limitations

Some limitations of this study should be taken into consideration. Firstly, the data was collected through self-report questionnaires, which may have been affected by social expectations and recall biases. Future studies could consider multi-informants, such as parents, teachers, and peers. Secondly, the generalizability of the results to Chinese adolescents in rural areas was limited by the sample size, as all students were recruited from only two schools in Guizhou and Sichuan in Southwest China. Moreover, given the voluntary nature of school participation, there is a possibility that schools with a greater interest in or commitment to mental health issues were more likely to participate. As a result, the schools involved in the current study may have a heightened awareness of mental health concerns, which may not reflect the broader spectrum of rural schools in China. Additionally, the use of convenience sampling was limited to schools supported by the foundation, which may restrict the generalizability of the findings to other rural areas not covered in specific areas. Another point worth noting is that the majority of participants in the current research lived on campus (94.5 %), which could also influence the applicability of the results. Future research should consider employing random sampling using a more diverse and representative sample (e.g., non-boarding school contexts) to mitigate potential biases and improve generalization. Thirdly, specific internet usage patterns (e.g., gaming vs. social media) and parental mental health were not included in this study, which may have influenced the observed associations. Fourth, future research should employ more research design methodologies to examine PIU. We only examined PIU profiles at one time point in the study. Future longitudinal designs are warranted to examine the temporal stability of PIU profiles and their long-term psychological impacts. Qualitative studies could further explore the lived experiences of the self-blame group, offering deeper insights into their unique challenges and coping mechanisms. Future research also should examine whether similar PIU profiles emerge in non-boarding school settings and other rural contexts.

## 6 Conclusion

In conclusion, this study sheds light on the latent profiles of PIU among rural Chinese adolescents, revealing four distinct subgroups with varying levels of severity across the three symptoms. The self-blame group emerged as a unique subgroup, demonstrating lower psychological distress despite higher PIU scores, likely due to their strong self-regulation. These findings highlight the heterogeneity of PIU behaviors and the critical role of cognitive and emotional factors. Risk factors for PIU include being female, being an ethnic minority, living off-campus, having left-behind experience, and having fewer siblings. Importantly, higher levels of PIU were associated with increased psychopathology, underscoring the importance of addressing internet obsession for rural Chinese adolescent mental health. Tailored interventions involving families and schools are crucial in mitigating the adverse effects of PIU among rural Chinese adolescents.

**Human ethics and consent to participate declarations**

All procedures performed in studies involving human participants were in accordance with the ethical standards of the institutional and/or national research committee and with the 1964 Helsinki declaration and its later amendments or comparable ethical standards. This study was reviewed and approved by the Ethics Committee of Chinese University of Hong Kong, Shenzhen (No. EF20220602002).

**Informed Consent**

Informed consent was obtained from all individual participants and their guardians included in the study.

## CRediT authorship contribution statement

**Yi Wang:** Writing – original draft, Data curation. **Brian J. Hall:** Supervision. **Yuran Chen:** Writing – review & editing. **Chun Chen:** Writing – review & editing, Supervision, Conceptualization.

## Funding

The study is funded by Pengcheng Peacock Matching Research Funding − Category C (No. 2024TC0135).

## Declaration of competing interest

The authors declare that they have no known competing financial interests or personal relationships that could have appeared to influence the work reported in this paper.

## Data Availability

Data will be made available on request.
